# Reducing social diabetes distress with a conversational agent support system: a three-week technology feasibility evaluation

**DOI:** 10.3389/fdgth.2023.1149374

**Published:** 2023-06-13

**Authors:** Merijn Bruijnes, Mitchell Kesteloo, Willem-Paul Brinkman

**Affiliations:** ^1^ Utrecht University School of Governance, Faculty of Law, Economics, and Governance, Utrecht University, Utrecht, Netherlands; ^2^ Department of Intelligent Systems, Faculty of Electrical Engineering, Mathematics & Computer Science, Delft University of Technology, Delft, Netherlands

**Keywords:** social diabetes distress, conversational agent, support system, personalised psych-education, self-help, longitudinal evaluation

## Abstract

**Background:**

People with diabetes mellitus not only have to deal with physical health problems, but also with the psycho-social challenges their chronic disease brings. Currently, technological tools that support the psycho-social context of a patient have received little attention.

**Objective:**

The objective of this work is to determine the feasibility and preliminary efficacy of an automated conversational agent to deliver, to people with diabetes, personalised psycho-education on dealing with (psycho-)social distress related to their chronic illness.

**Methods:**

In a double-blinded between-subject study, 156 crowd-workers with diabetes received a social help program intervention in three sessions over three weeks. They were randomly assigned to receive support from either an interactive conversational support agent (n=79) or a self-help text from the book “Diabetes burnout” as a control condition (n=77). Participants completed the Diabetes Distress Scale (DDS) before and after the intervention, and after the intervention, the Client Satisfaction Questionnaire (CSQ-8), Feeling of Being Heard (FBH), and System Usability Scale (SUS).

**Results:**

Results indicate that people using the conversational agent have a larger reduction in diabetes distress (M=−0.305, SD=0.865) than the control group (M=0.002, SD=0.743) and this difference is statistically significant (t(154)=2.377, p=0.019). A hypothesised mediation effect of “attitude to the social help program” was not observed.

**Conclusions:**

An automated conversational agent can deliver personalised psycho-education on dealing with (psycho-)social distress to people with diabetes and reduce diabetes distress more than a self-help book.

**Ethics, Study Registration and Open Science:**

This study has been preregistered with the Open Science Foundation (osf.io/yb6vg) and has been accepted by the Human Research Ethics Committee - Delft University of Technology under application number 1130. The data and analysis script are available: https://surfdrive.surf.nl/files/index.php/s/4xSEHCrAu0HsJ4P.

## 1. Introduction

In this work, we present and evaluate a conversational agent that can help people with diabetes to deal with various, socially related diabetes distresses. The number of people suffering from diabetes is growing at an alarming rate, rising from 108 million in 1980 to 463 million in 2010 globally (1). Diabetes is a chronic disease where the body either does not produce enough insulin, a hormone that regulates blood sugar levels, or where the cells are not sensitive to the hormone. The disease can cause many complications such as kidney failure, loss of vision, heart or brain stroke and nerve damage. To reduce the severity of these complications, a person with diabetes (PWD) will have to check their blood sugar levels and take medication depending on their body’s sensitivity to insulin and insulin production. Additionally, people with diabetes are advised to regulate their diet and, depending on the type and severity of their diabetes, change other aspects of their lifestyle (2).

Adopting a healthy lifestyle and managing diabetes often means changing deep-seated behaviour patterns which are very difficult without the right support. In fact, social support from family members and peers has been shown to be crucial in maintaining lifestyle changes and optimising diabetes management (e.g. (3–6)). It is therefore unfortunate that diabetes is surrounded by social stigma and that symptoms and management of diabetes often elicit adverse social reactions. For example, a patient having hypoglycemia (blood glucose level too low) can exhibit behaviour such as slurred speech or clumsiness which can be mistaken for being drunk (7). Alternatively, managing diabetes can require drawing blood to measure blood sugar levels or injecting insulin, which in public spaces can be mistaken for drug abuse and draw unwanted attention. The (expected) judgement or unwelcome interest from the environment can negatively affect the psychological well-being and even therapy adherence of the person with diabetes (8). Closer to home, concerned partners or parents can be overly protective (7) or misunderstand the needs of the patient (9). This is often a source of conflict in the close social environment and can lead to a perceived overabundance or lack of social support. Problematic social support adds additional stress to a PWD who, already distressed by having to deal with their disease, now also feels the burden of having to manage their social support circle (10–12). This diabetes distress is observed for people with type-I (13) and with type-II diabetes (14).

Physicians determine whether a patient has problems with diabetes distress using instruments like the Problem Areas In Diabetes (PAID) scale (15, 16) or Diabetes Distress Scale (DDS) (11), and if needed they recommend mental health therapy. However, the social stigma surrounding mental health is a barrier that prevents many patients to follow up with therapy leaving the distress untreated (17). Alternatively, patients can be pointed towards websites of diabetes organisations (e.g., (18–20)) or towards self-help books (e.g., (11)) for advice on how to deal with diabetes distress. Unfortunately, these sources are often focused on delivering general information about diabetes and have limited information on how to deal with (social) diabetes distresses. Additionally, such self-treatment is not personalised or structured by a therapist to the specific need of the PWD. Thus, currently, PWD suffering from (social) diabetes distresses are not reached by medical professionals or have to resort to sub-optimal self-treatment.

We propose a conversational agent that can give the PWD the information they need to address their problem: an intervention personalised to the situation of the PWD. A conversational agent can explore the problem together with the PWD and then tailor its advice to the situation. Conversational agent interventions have less stigma and a lower barrier to start than traditional mental healthcare because, for example, the interactions can be anonymous or “not with a human” (21). This means that more people might be perceptible to receive the care they need if it is given by a conversational agent. Conversational agents have shown to be effective in the mental health domain, for example, by delivering cognitive behaviour therapy to people with symptoms of depression and anxiety (22) or by using elements of motivational interviewing and social cognitive therapy to promote exercising and a healthier diet to users (23). Many other examples of conversational agents in health exist, for example, conversational agents used for applying therapy, self-management, intervention and counselling successfully (see for an overview (24, 25)). Recently, mobile app-based interactive conversational agents to support people with type-2 diabetes have been discussed, showing that the self-management education such systems offer can be accepted by the PWD (26) and that such systems can be effective in improving the reported health-related quality of life of the PWD (27). However, without claiming to have conducted a comprehensive literature review, we are unaware of studies on conversational agent systems specifically supporting PWD with social diabetes distress.

In this paper, we investigate the feasibility of a conversational agent that, over multiple sessions, determines a PWD’s social diabetes distress and gives appropriate tips to reduce this distress. When this technical intervention is found feasible it might be considered in a broader diabetes support intervention including, for instance, sessions with a (mental) health professional and support with monitoring and managing health. Such a broader health intervention should be tested in a randomised control trial (RTC), however, this is not within the scope of this paper. To ascertain the feasibility of our technical intervention, we compare the outcomes of a three-week social help program consisting of either interactions with our conversational agent (chatbot) or an information control group (self-help book). We hypothesise that people using the conversational agent have a larger reduction in diabetes distress than the control group (H1) because a conversational agent can interactively personalise the information provided. The personal preference for a social help program might influence the effectiveness of the intervention, thus, we expect that the effect of H1 is mediated by the attitude to the social help program (H2). Further, following the logic that people with low diabetes distress have “less room” to improve than people with higher distress, we hypothesise that people who have higher initial diabetes distress have a larger reduction in diabetes distress than people with lower initial diabetes distress (H3). Finally, we hypothesise that people using the conversational agent have a more positive feeling of being heard than the control group (H4), because of the interactivity of a conversational agent and the personalised information provided.

## 2. Methods

### 2.1. Participant recruitment

Participants were recruited through the online crowd-worker platform Prolific.^1^ Recently, Jonell et al. (28) showed that crowd-workers produce results similar to lab participants under observation, indicating that using crowd-workers is acceptable. Prolific offers pre-screening of participants, including (self-reported) diabetes, making using crowd-workers also opportune for fast and large-scale research with specific groups like PWD. A sample size estimation for two independent groups assuming a distribution-free test showed a minimum required sample size of 134 participants: effect size d=0.5 (a medium effect according to Cohen (29)), error probability α=0.05, power 1−β=0.80 and asymptotic relative efficiency ARE=0.955 (30). The participant selection criteria were self-reported diabetes (type-I, type-II, or other) and English proficiency. Participant exclusion criteria were failed attention checks and not completing all sessions. Recruitment was continued in batches until the sample size requirements were met with similar numbers of participants in the two conditions. Participants were paid a minimum of 6 GBP/h according to the platform’s norms with an increasing bonus for completing consecutive sessions to reduce attrition.

### 2.2. Intervention: social help program

Participants were randomly assigned to a social help program (the control group or the agent group) through a double-blind between-subjects design. Existing psychological interventions aimed at helping PWD deal with diabetes distress are longitudinal (e.g., (31–35)) so that in a next session the success or failure of an intervention to reduce distress can be addressed. Additionally, multiple sessions give the opportunity for experiential learning (36). Our intervention consisted of three sessions for both conditions. In the sessions, the conversational agent iterates over providing advice, evaluating the usefulness of the previous advice and giving alternative advice, or working on another issue, while the control group (re)reads a self-help text.

For both conditions in our intervention, we focus on two common social diabetes distresses: interpersonal distress and friend/family distress (11). Interpersonal distress can be characterised as receiving too little support, while friend/family distress can be characterised as receiving too much support and is often referred to as “diabetes police.” The book “*Diabetes burnout: What to do when you can’t take it anymore*” by Polonsky (11) provides tips and strategies to deal with both distresses and is used as the basis for the social help program.

#### 2.2.1. Conversational agent

The design of the agent is based on existing “analogue” psychological interventions for PWD (31–35) and contains elements of *shared decision making* (37). Most of these interventions start with determining the PWD’s problem and then giving tailored advice to deal with that specific problem. Additionally, these interventions aim to actively engage the participant by, for example, asking them to come up with potential solutions to their problem and discussing the advantages and disadvantages of each solution. Such active engagement in deciding which solution to pursue makes patients more likely to follow through (37).

**Figure 1:**
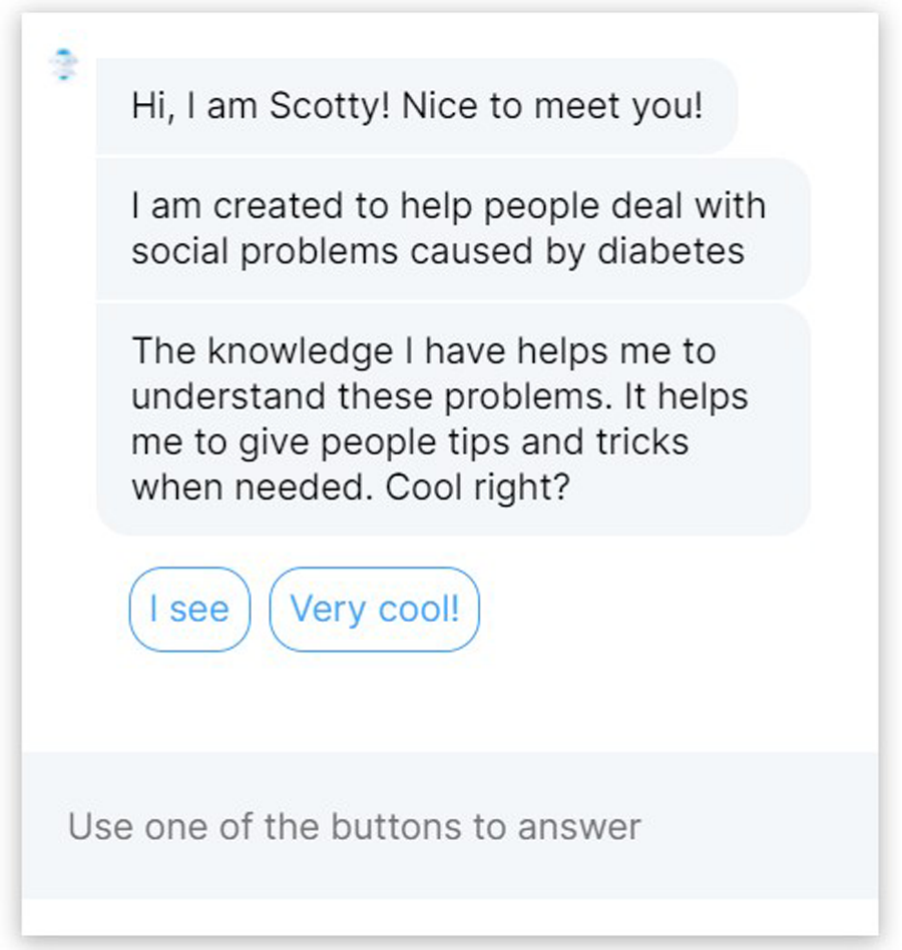
Interface of the conversational agent, showing the start of the first session.

**Figure 2:**
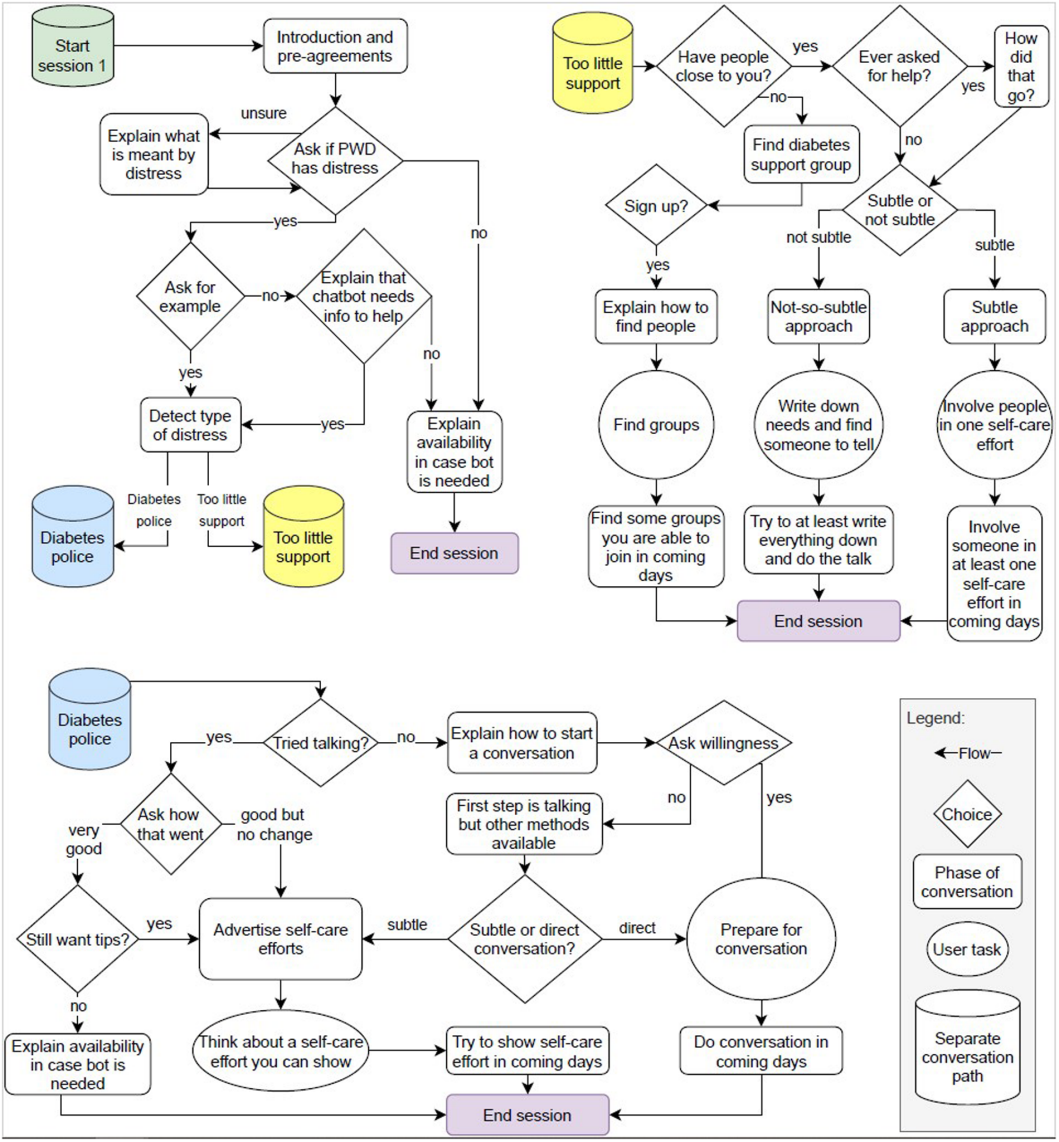
The conversation flow of the first session of the conversational agent.

The conversational agent starts every session explaining its goal for the current session (see Figure 1), following Kretzschmar et al. (38) and Bickmore et al. (39) who state that users should know what an agent targets and how it tries to achieve its goal. Next, the agent determines which social diabetes distress is most problematic for the user and presents strategies that are appropriate to deal with that specific distress. The strategies come from the book “Diabetes Burnout” by Polonsky (11) and are the same as for the control condition. The agent discusses the pros and cons of the most appropriate strategies with the user. The user is asked to make a choice for their preferred strategy. The agent then presents the purpose of that strategy in more detail to establish trust in the strategy and in the agent (40). The information the agent presents is a subset of the content in the control condition, as only the strategy selected by the user is presented in detail. Finally, the agent ends the session (see Figure 2). In the next sessions, the agent and user reflect on whether the strategy worked and, if not, whether another strategy should be explored, or whether another distress should be addressed. Beyond these differences, the subsequent sessions follow the same structure and content as the first session.

The technical implementation of the conversational agent utilised the Rasa toolkit (41). Rasa provides a complete architecture where conversational agent creators can quickly roll out a text-based conversational agent: a chatbot. The architecture includes a Natural Language Understanding (NLU) pipeline, dialogue management, Rasa X (a back-end with GUI and the ability to connect to a Git repository), the ability to create custom I/O channels, a custom action server, and several other services like event and conversation trackers. An interaction over multiple sessions means the state of the conversation has to be saved, for which we used the lightweight database management system Sqlite,^2^ and linked to a particular participant for which we used the Prolific ID of the user. The user interface was created using the Rasa Webchat^3^ widget, see Figure 1. Participants could type in the text box at the bottom of the page or, when available, use buttons with dialogue options to advance the dialogue. However, note that the state transitions in the conversation flow (Figure 2) were prescripted (only those transitions are possible) and could be made through free text input or if that failed through buttons which reflected the options available. For example, to detect diabetes distress, the agent asked the participant to give an example of a social issue they experienced recently. The agent then determines the diabetes distress from the free text using the Rasa NLU pipeline. The pipeline was trained with about 50 example stories of each distress, which were obtained from PWD and people who know a PWD. To ensure accurate recognition and consequently appropriate advice, the agent asked the participant for verification of the recognised distress. If the distress was not recognised correctly, the user was asked to elaborate on their example situation. If it was still not recognised correctly, the agent presented buttons with diabetes distresses that it knows about. Finally, for “simple transitions” (e.g., yes/no) buttons were presented and no free text input was allowed (see also Figure 1).

#### 2.2.2. Control condition

The book “Diabetes Burnout” by Polonsky (11) contains tips for people with diabetes on how to deal with the different forms of diabetes distress. For example, the book gives readers strategies to assertively manage challenging social situations, for educating the close social environment, and on how to organise support for oneself. Participants in the control condition read the same text in each session as their intervention, specifically (11, p. 215–245), that deal with interpersonal distress and friend/family distress.

### 2.3. Measures

To measure *social diabetes distress*, we used a combination of the type-1 diabetes distress scale (14) and the type-2 diabetes distress scale (13). Specifically, we used the interpersonal distress, friend/family distress and negative social perceptions distress sub-scales from the type-1 Diabetes Distress Scale (14) and type-2 Diabetes Distress Scale (13) surveys. The questions are on a scale from 1 to 6, where a higher score means higher distress and a lower score means lower distress. The original questionnaires contain an instruction asking to answer the questions based on what the PWD has experienced in the past four weeks. However, as our intervention spans only three weeks, we changed the instruction to what the participant expects to experience in the coming 4 weeks.

The *attitude towards the intervention* was measured using the Client Satisfaction Questionnaire (CSQ-8) (42), consisting of 8 items scored on a scale from 1 to 4. The sum of scores on all items gives the final score (ranging from 8 to 32).

The *Feeling of Being Heard* (FBH) (43) questionnaire was used to measure whether the type of social help the participant receives influences the feeling of being heard. The FBH questionnaire consists of 7 statements. In Tielman et al. (43), the questions were answered by using a scale where participants could indicate how much they agreed to a statement by clicking a point on a continuous scale from “it decreased a lot” to “nothing changed” in the middle of the scale to “it increased a lot.” However, even though a continuous scale gives more answer options to the participant, it also makes it harder for the participant to determine which response option comes closest to their actual opinion (44). Additionally, the reliability and validity of responses to questions increase with more response options, but this increase levels off after providing 7 response options (45). Therefore, we used a 7-point Likert scale. The scores are averaged to get one final score.

The System Usability Scale (SUS) was used to check whether the implementation of the conversational agent was sufficiently usable. We consider the implementation to be sufficient if the average SUS falls at least in the “OK” category (a score of 50.9 with a standard deviation of 13.8) as described by Bangor et al. (46).

### 2.4. Procedure

The experiment consisted of three sessions, for both intervention conditions, and each session was separated by at least one week. Participants received an automated email invitation for the next session seven days after completing the previous session. In the first session, participants gave informed consent, filled in the diabetes distress questionnaire (pre-intervention measure), and then were redirected to the intervention (either the conversational agent or the self-help text condition). In the second and third sessions participants again received their intervention. After the intervention in session three, participants filled in the diabetes distress questionnaire (post-intervention measure), the CSQ-8 to measure the attitude towards the social help program, and the feeling of being heard questionnaire. Additionally, participants in the conversational agent group filled in the System Usability Scale. Finally, each session ended with an optional open question where participants could give any comments they might have.

### 2.5. Analyses

**Figure 3:**
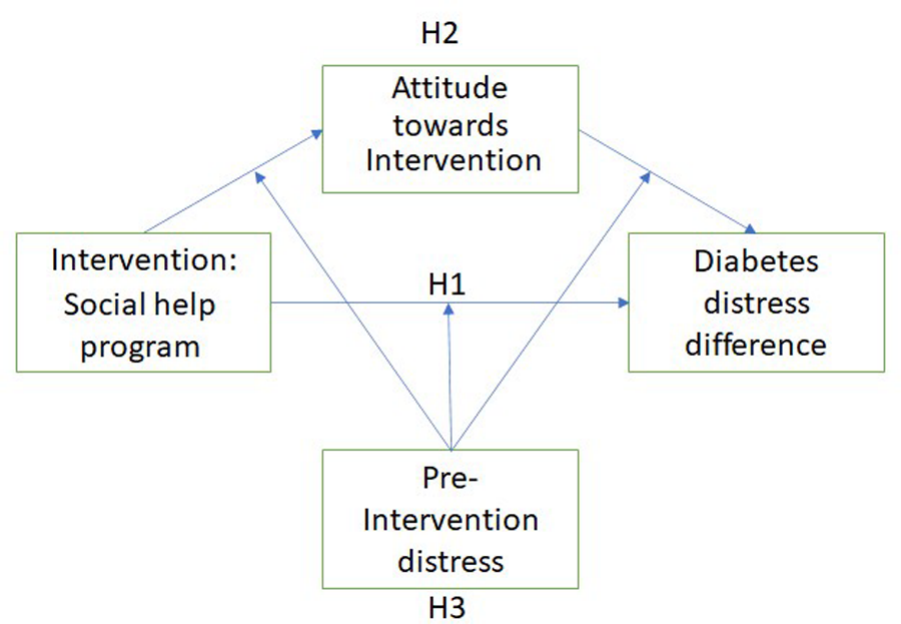
Conceptual model: hypothesised influences on the diabetes distress difference.

Summarising our conceptual model, see Figure 3, we compared the diabetes distress before and after the intervention. We compute this *diabetes distress difference* by subtracting the diabetes distress score before the intervention from the score after the intervention. We used a two-sided, two-sample t-test to test whether the conversational agent caused a larger reduction in diabetes distress than the control group (H1) and whether the conversational agent resulted in more feeling of being heard than the control group (H4). To test whether the effect of the interventions on the diabetes distress difference is mediated by the attitude towards the intervention (H2) we used the bootstrapping method of Preacher and Hayes (47) to estimate the mediating effect over 1000 random samples. For this method, data does not have to be normally distributed and, according to Preacher and Hayes, this method addresses the power limitations of the standard Sobel test. We looked at the Average Causal Mediation Effects (ACME), which is the indirect effect of the mediator (total effect - direct effect) and shows whether the influence of the mediator is significant. A negative coefficient means that the mediator causes a decrease in diabetes distress. To test whether people with higher initial diabetes distress have a larger reduction in diabetes distress than people with low initial diabetes distress (H3), we performed a moderation effect analysis by computing the interaction between pre-intervention diabetes distress and the intervention type. We fitted a linear model where diabetes distress difference is explained by both variables and their interaction, and checking the significance of the coefficient of the interaction term. Furthermore, we did not centre the independent variable and moderator since centring, with the exception of cases of extreme collinearity, does not make any difference in testing the interaction term (48). When the assumptions of multiple linear regression models (such as the assumption of a linear relationship between independent and dependent variables) were not met, appropriate steps were taken. Finally, the data and the R-markdown script of the analyses are available.^4^ Finally, the first and second authors performed a qualitative analysis of the comments participants gave about the conversational agent at the end of each session, by labelling positive and negative comments and defining the themes (49). The second author made the initial labelling, which was checked by the first author. Different labelling was discussed until an agreement was reached.

### 2.6. Ethics, informed consent and privacy

The study has been approved by the data management officer and the Human Research Ethics Committee of the Delft University of Technology under application number 1130 and found in accordance with privacy legislation. All participants gave informed consent before participation could revoke their consent and have their data removed during and for a limited time after the study. Conversational agent data was stored on a secured and access-restricted university server. Questionnaire data was stored on a Qualtrics server in the EU. Participants were known only by their anonymous crowd-platform ID which was removed and replaced with a random identifier after study completion. All (pseudo-anonymous) data was irreversibly made anonymous before analyses by the second author. The study was preregistered with the Open Science Foundation.^5^

## 3. Results

### 3.1. Participants

We recruited English-speaking adults with diabetes via the crowd-worker platform Prolific. Participants received a monetary payment for their time (averaging 8.40 GBP/h). We suffered a high exclusion and attrition rate (37.3%) due to failed attention checks and failure to return for all sessions. We continued recruitment in batches until the sample size requirements were met and the groups had similar sizes. Every batch of participants finished the experiment—with at least seven days between the three sessions—in about three weeks as most participants did not respond immediately to the invitation to the next session. The first batch ran between November 24th–December 14th 2020; the second between December 7th–December 30th 2020; and the last between January 13th–January 29th 2021. Eventually, the data from 156 participants could be used in the analyses. Participants were randomly assigned to receive support from either our interactive conversational support agent (n=79) or a self-help text as a control condition (n=77). The participant demographics were gender (male=82), age (mean=28.6, SD=15.6), and pre-intervention diabetes distress (mean=2.6, SD=1.1), see Table 1.

**Table 1:** Profile of the participants.

Participants	Control group	Treatment group	Total
Number, n	77	79	156
Male, n(%)	48 (62.3%)	34 (43%)	82 (52.6%)
Age (years)			
Mean (SD)	40.3 (16.3)	37 (14.9)	38.6 (15.6)
Range	18–76	18–70	18–76
Pre diabetes distress			
Mean (SD)	2.4 (1)	2.7 (1.1)	2.6 (1.1)
Range	1–5.4	1–5.1	1–5.4

### 3.2. Pre-trail bias checks

We investigated whether there were any significant differences between the groups regarding age, gender and pre-intervention diabetes distress using a Kruskal-Wallis test. There was a significant difference between genders in the control and agent group ($\chi^{2}(1)=5.788$, p=0.016). We checked whether this difference had any influence on the results by fitting a linear model where the diabetes distress difference is explained by the gender of the participant. The gender coefficient was not statistically significant (p=0.863), implying that this variable did not influence the results.

We used the System Usability Scale to determine whether the implementation of the conversational agent was sufficiently usable, for which at least an average score of 50.9 with a maximum standard deviation of 13.8 is needed (46). The mean score is 81.6 (SD=12.0), which can be interpreted as “Good” or “Excellent” usability.

### 3.3. Hypotheses testing

#### H1: People using the conversational agent have a larger reduction in diabetes distress than the control group

Comparing the diabetes distress difference of participants in the agent group (M=−0.305, SD=0.865) with the participants in the control group (M=0.002, SD=0.743), we see that there is a statistically significant difference in diabetes distress, t(154)=2.377, p=.019. For the agent group, the diabetes distress difference was statistically significantly lower than zero indicating a reduction in diabetes distress (one-sided, one sample: t(78)=−3.133, p=0.0012) while for the control condition, it was not (p>0.5). The effect size (d=0.38) is between small and medium according to Cohen’s convention (29).

#### H2:The effect of the intervention type on the diabetes distress difference is mediated by the attitude towards the intervention

The mediated (i.e., indirect) effect of the intervention via the attitude towards the intervention on the diabetes distress difference, the ACME, is not statistically significant (est.=0.0012, 95%CI=[−0.0292,0.04], p=0.872). Thus, there is no mediation effect.

#### H3: People who have higher initial diabetes distress have a larger reduction in diabetes distress than people with low initial diabetes distress

To analyse whether initial distress moderates the reduction in distress, we fitted a linear model where the diabetes distress difference is explained by the intervention, the diabetes distress before exposure to the intervention and their interaction. Moderation would be present when the coefficient of the interaction term is statistically significant. It was not: t(154)=−1.794, p=0.075.

#### H4: People using the conversational agent have a larger feeling of being heard than the control group

Participants in the agent group (M=4.79, SD=0.84) and the control group (M=4.70, SD=1.02) do not differ statistically significantly for the feeling of being heard (t(154)=−0.60, p=0.55).

### 3.4. Qualitative results

**Figure 4:**
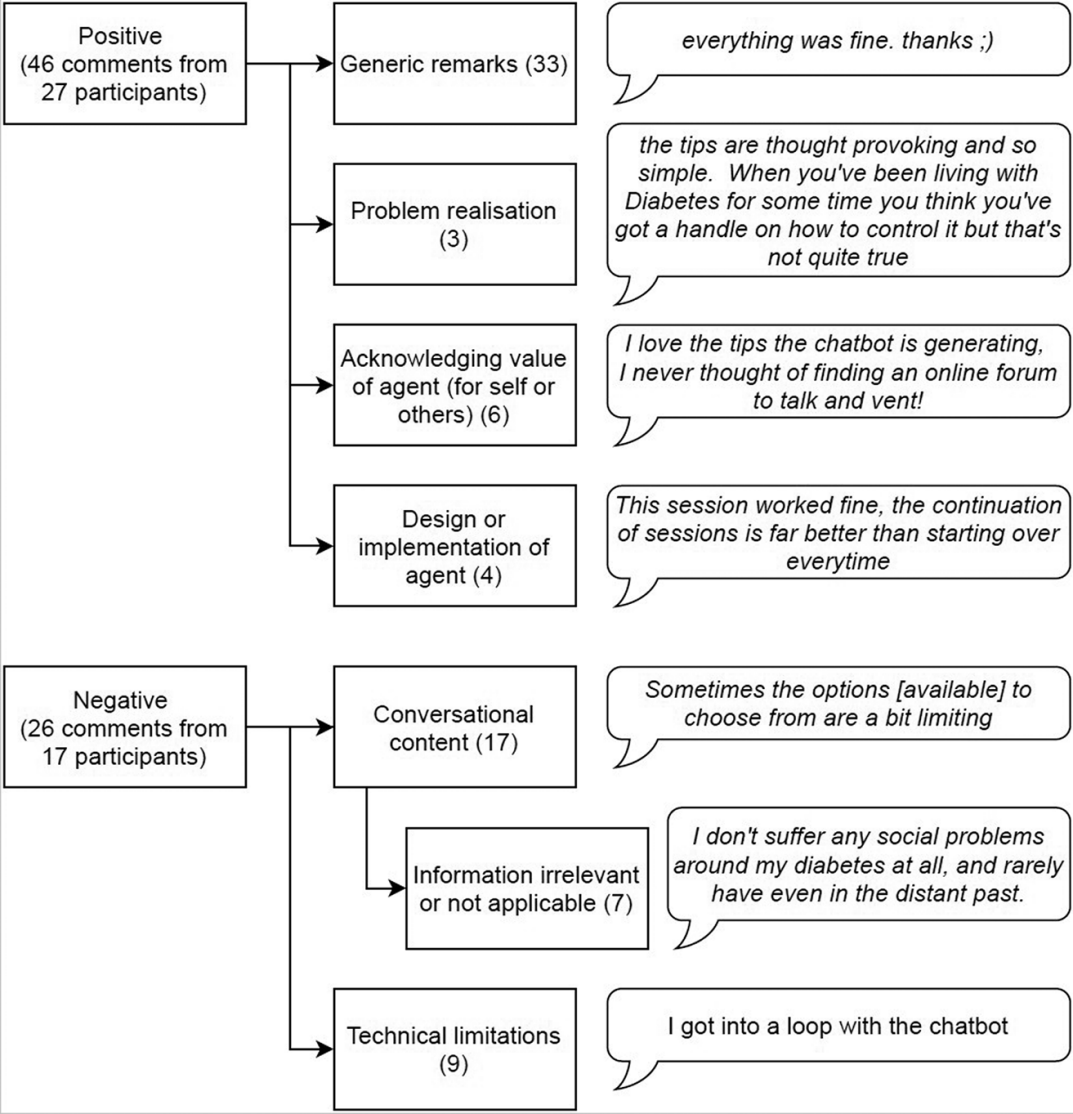
Thematic map of participants’ comments about the chatbot.

he thematic map of participants’ comments about the chatbot, see Figure 4, shows that from the positive responses (n=46) most were generic positive comments (n=33). Other positive themes that emerged were participants realising that they had a social diabetes distress problem (n=3), acknowledging the value of the chatbot (n=6), or commenting positively about the design or implementation of the intervention (n=4). Negative comments (n=26) were divided in two themes: comments about the limited conversational content of the chatbot (n=17) and technical issues or limitations (n=9). We defined, as a sub-theme of the limited conversational content, comments that mentioned specifics about the information that the chatbot provided that was not relevant for the participant or not applicable to their situation (n=7).

## 4. Discussion

Our findings show the feasibility of a conversational agent determining a PWD’s social diabetes distress and giving appropriate tips to reduce the distress (*H1*). This adds evidence to the notion that the interactivity of a conversational agent and personalised advice is indeed important (43, 50). Furthermore, our finding adds social diabetes distress to the long list of problems that a conversational agent can potentially address (e.g. (24, 25)). Investigating a potential reason underlying the positive effects of a conversational agent as an intervention, we hypothesised that the attitude of the participant towards the intervention (either the conversational agent or the self-help text) might have a mediation effect on diabetes distress difference (*H2*). We did not observe this mediation effect. Thus, in our case, satisfaction with the intervention did not influence its effectiveness. However, the scale used, the CSQ-8, is intended for evaluating patient satisfaction with (mental) healthcare services received from humans and as such has not been validated for conversational agent satisfaction. Another reason the conversational agent might be more effective than a self-help text is that the agent can elicit a feeling of being heard (*H4*) due to its interactivity and personalisation (e.g. (43)). However, we did not observe a significant difference between the two conditions in the feeling of being heard despite that the agent condition was more successful than the control condition. Our approach is different than the work by Tielman et al. (43): where they send personalised motivational messages based on the situation (progression of PTSD symptoms and the user’s trust in positive therapy outcome), we provide personalised information based on the type of social diabetes distress of the participant. A conversational agent that does consider the progression of the diabetes distress and the user’s trust in strategies and tips provided might be able to elicit feelings of being heard, which in turn might increase the positive effect that we observed. Finally, the diabetes distress before exposure to the invention did not moderate the effect of the intervention on the diabetes distress difference (*H3*).

Looking at the comments participants gave at the end of the intervention and exploring these through the lens of the trans-theoretical model of behaviour change (51), provides clues for who this conversational agent might be most beneficial. The trans-theoretical model posits that a person’s readiness to adopt new behaviour involves six stages of change: pre-contemplation, contemplation, preparation, action, maintenance, and termination. We observe that the majority of comments are positive. However, most of the positive comments are generic remarks, such as “*everything was fine, thanks*,” that hold little information. Our conversational agent started the intervention with an exploration of diabetes distress problems that a user might have. Some users commented that this approach was successful for them, as they realised which problems they were (still) facing. Thus, a conversational agent might play a role in the process of raising conscious awareness of the problem (i.e., for participants who are in the pre-contemplation stage). Additionally, some users commented that they saw the value of the agent in giving concrete strategies to deal with their issue, indicating the agent facilitates the process of preparation to deal with their problem (i.e., for participants in the contemplation and preparation stages). Considering the observed reduction in diabetes distress, the conversational agent might have played a role in activating users to address their problem (i.e., for people in the action stage). Additionally, despite that the intervention lasted about three weeks, that the diabetes distress was reduced after this period, and that participants commented positively about this longitudinal setup, it remains an open question whether lasting behaviour change was achieved and thus whether the agent played a role in the maintenance stage of behaviour change. In conclusion, we argue that this type of conversational agent intervention might be opportune in each stage of behaviour change. Finally, we are aware that the observed diabetes distress reduction might be explained by alternative processes. For example, the distress might be due to expected social problems for which it might have been effective to raise the user’s self-efficacy (i.e., the belief that one can deal or cope with their problems) (52) without changing any behaviours.

### 4.1. Limitations

Our intervention duration was relatively limited with three separate sessions over a three-week period and a longer intervention (of months or a year) or more sessions might increase the effect size. However, other conversational agent interventions, for example, reducing depression (22), managed comparable effect sizes with a similar intervention duration. A longer intervention would require a conversational agent to have more conversational content to not “bore” the user. This could, for example, be achieved with the ability to address more types of diabetes distress and have more strategies to explore for each type of diabetes distress. Further, we have not performed a follow-up to determine whether the reduction of distress was persistent over a longer period after the intervention. However, we argue that the current study duration was suitable for this technology feasibility study: now we know this type of intervention can reduce social diabetes distress. As this was not known before, we could not defend the (substantial) additional expenses for a longer study. With our findings in hand, future work, such as an RTC, should determine whether this distress reduction is sustainable over longer periods.

The relatively high attrition rate, 38% of participants failed attention checks or stopped in between sessions, might indicate a problem with the intervention. However, attrition was similar in both conditions and as such is likely not related to the conversational agent. Potentially, the topic addressed in the intervention, diabetes distress, was confrontational to some PWD which might have contributed to drop-outs. However, the topic was explicitly and thoroughly explained in the recruiting text on the crowd-platform and in the informed consent. Thus, the crowd-workers for who this would have been an issue likely opted not to participate. Another potential cause for the high attrition is that we did not pre-select people with a high approval rating on prior crowd-studies, which is a good predictor of the quality of a participant’s submission (e.g., passing attention checks). We opted not to do this since there was a limited number (n≈2000) of eligible participants (i.e., with self-reported diabetes) available on this platform and we did not want to reduce the potential pool of participants further as a (much) smaller pool might threaten the anonymity of participants (e.g., (53)). Future studies might explore additional sources, such as other crowd-platforms or diabetes clinics, to find a larger pool of eligible participants.

Finally, we did not collect information about the participants’ type of diabetes nor whether participants currently receive therapy for diabetes distress. The ethics committee advised against collecting medical data that is not necessary to answer the hypotheses. Therefore, it remains an open question whether the type of diabetes may have an effect on the influence of the agent on the diabetes distress difference. Furthermore, looking at the age distribution, it is unlikely that the ratio of type-I and type-II diabetes in our participants reflects the ratio in the general population. Roughly 90% of all people with diabetes suffer from type-II, but this is often diagnosed at later ages (after 45) (54) while most of our participants were younger (M=38.6, SD=15.6). Similarly, we collected no information on whether the participant is currently in therapy for diabetes distress and therefore it is not possible to investigate the potential confound of such therapy. However, now that our work showed the potential for a conversational agent intervention to reduce diabetes distress, future research might want to include these factors. For this, we recommend explicitly considering the balance of power in the relationship between the PWD and the research group, for example, avoid a situation where participants (believe that they) will be disadvantaged if they do not consent to share their medical data, for example, because they will not receive the potentially beneficial therapy.

### 4.2. Conclusion

We investigated whether a conversational agent could, over multiple sessions, determine a PWD’s social diabetes distress, give appropriate tips and reduce the distress: it could. Compared to a control group that (re)read a part of a self-help book, the conversational agent performed better with regard to reducing diabetes distress. Our finding shows conversational agents’ potential to improve the lives of the millions of people with social diabetes distress that are currently not targeted or reached by traditional healthcare: a sweet future to look forward to.

## Data Availability

The dataset and material is available here: https://surfdrive.surf.nl/files/index.php/s/4xSEHCrAu0HsJ4P. Requests to access the datasets can be directed to the first author.

## Footnotes

^1^ https://app.prolific.co/

^2^ https://www.sqlite.org/

^3^ https://github.com/botfront/rasa-webchat

^4^Data and analyses materials: https://surfdrive.surf.nl/files/index.php/s/4xSEHCrAu0HsJ4P

^5^View the preregistration here: https://osf.io/yb6vg
